# Detection of Low Adherence in Rural Tuberculosis Patients in China: Application of Morisky Medication Adherence Scale

**DOI:** 10.3390/ijerph14030248

**Published:** 2017-03-01

**Authors:** Minlan Xu, Urban Markström, Juncheng Lyu, Lingzhong Xu

**Affiliations:** 1 School of Public Health, Shandong University, Jinan 250012, China; lzxu@sdu.edu.cn; 2 Department of Social Work, Umeå University, 901 87 Umeå, Sweden; urban.markstrom@umu.se; 3 Department of Public Health, Weifang Medical University, Weifang 261042, China; cheng_china@163.com

**Keywords:** tuberculosis, medication adherence, C-MMAS-8, reliability, validity, associated factors

## Abstract

The detection and analysis of cases of low medication adherence is important for helping to control tuberculosis (TB). The purpose of this study was to detect low adherence in rural TB patients by using the eight-item Morisky Medication Adherence Scale of Chinese version (C-MMAS-8) and to further analyze the adherence-related variables. A total of 358 rural TB patients recruited through multi-stage randomized sampling participated in the survey. Data were collected by the use of interviewer-led questionnaires. First, the reliability and validity of the C-MMAS-8 were determined. Second, the adherence level was assessed, and factors related to low adherence were analyzed by using Pearson’s chi-square test and then in multiple logistic regression model. Finally, the prediction of the logistic model was assessed with Receiver Operating Characteristic (ROC) curves. The C-MMAS-8 could be used to detect low adherence in TB patients with good reliability and validity. By using the referred cutoff points of MMAS-8, it was found that more than one-third of the participants had low medication adherence. Further analysis revealed the variables of being older, a longer treatment time, and being depressive were significantly related to low adherence. The ROC of the model was assessed as good using the cutoff point. We conclude that appropriately tailored strategies are needed for health-care providers to help rural TB patients cope with low medication adherence.

## 1. Introduction

Medication adherence can be defined as the extent to which the patients take medications as prescribed by heath care providers and as agreed upon in the patient’s treatment plan [1]. Tuberculosis (TB) is a chronic infectious disease that can be transmitted via droplets from patients with active pulmonary tuberculosis, and its treatment involves a 6–9-month medication regimen with combinations of drugs, which may cause side effects and non-adherence. Low level of adherence to medication is cited as one of the most important barriers to TB control [2]. Poor adherence to TB medication can be particularly problematic because it can result in prolonged treatment, higher costs, an increase in new cases, and the development of multidrug resistance. These outcomes make treatment more complex and more expensive. China is among the 22 countries with the highest burden of TB despite the great progress that has been made in controlling TB in the past decades [3]. There is an estimate that 80% of registered TB patients live in rural areas where there is a lack of healthcare resources [4]. The DOTS (Directly Observed Treatment Strategy), a preventive strategy for non-adherence, has spread countrywide, yet it is not efficiently implemented in many areas [5,6]. A study found that TB control programs did not fully function in remote and rural areas due to poor treatment adherence in China [7]. Therefore, the study of medication adherence and detection of its related factors are important for controlling TB in rural areas.

There have been many approaches to measure medication adherence such as patient self-reports, pharmacy documents, tests for biomarkers, and electronic monitoring devices. For rural TB patients, a cheap and convenient way of screening adherence is to use scales. The eight-item Morisky Medication Adherence Scale (MMAS-8) is one of the simplest self-report scales measuring medication adherence behavior [8]. The reliability and validity of the MMAS-8 are being or have already been measured in other languages across different populations, settings, and diseases [8,9,10,11,12]. The Chinese version of the MMAS-8 (C-MMAS-8) was stepwise processed with translation and back-translation according to international guidelines and was validated in an adherence study with myocardial infarction patients [13], but not yet in TB patients.

A systematic review of qualitative research on patient adherence to TB medication concluded that a wide range of interacting factors impact on medication-taking behaviors, and person-related factors were found to be one of the main reasons for lack of adherence [14]. Therefore, the aims of this study were (1) to validate the C-MMAS-8 in its first application with rural TB patients and (2) to use the C-MMAS-8 to measure levels of low medication adherence and to analyze the factors that determine a patient’s low adherence in medication. The results could provide healthcare providers and policy-makers with strategic clues for improving medication adherence in rural TB patients.

## 2. Methods

### 2.1. Sampling and Settings

A cross-sectional survey was carried out in rural areas in Shandong Province, which is located in Eastern China and has a population of nearly 100 million, rich in the east and poor in the west. By using multi-stage random sampling, three regions based on the economic level were first sampled from the western, middle, and eastern provincial areas. Then, in each region two counties were selected, and in each county six rural towns were randomly selected. Next, 36 towns in six counties were chosen as the survey sites. Finally, patients were cluster-sampled from those selected towns, where all the eligible TB patients were included and represented samples for the study. In China, county TB dispensaries (CTD) are the official institutions in rural areas responsible for TB diagnosis, treatment, public health service, and case management including registration of newly diagnosed TB patients and dispensing free anti-TB medication monthly. The free treatment policy covers only the costs of first-line anti-TB drugs and excludes the costs of hospitalization, second-line anti-TB drugs, and any other drugs. Therefore, in this study hospitalized patients, recurrent cases after failed treatment, and drug-resistant cases were not included. Next, with the help of local CTD staff, eligible TB patients being treated at home were recruited and household visits were arranged through telephone calls. For the TB patients, structured face-to-face interviews were undertaken in the form of interviewer-led questionnaires; each interview took about one hour. The interviewers consisted of institute staff and graduate students, all of whom received standard training by attending a workshop to study the survey protocol and guidelines for collecting data. The CTD staff helped to organize and supervise the field survey.

### 2.2. Questionnaire and Definition of Variables

The questionnaire was developed based on literature reviews [8,13,15] and expert consultations, and consisted mainly of sociodemographic characteristics, clinical data, psychological status, and medication adherence. Sociodemographic characteristics included age, gender, living with partner (yes/no), school years, and self-reported household income level (high/middle/low), while clinical data were presented in the form of treatment length and costs out of one’s own pockets, self-rated illness severity (no/minor/moderate/severe), and limitations on daily life (no/minor/moderate/severe). Psychological status was measured with the Kessler Psychological Distress Scale (K10), which is a five-point Likert scale with 10 items measuring anxiety or depression and other psychological-related symptoms in the past four weeks [16]. The Chinese version of K10 showed a good level of consistency with the Symptom Checklist-90 (SCL-90) [17]. On the basis of the K10 cutoff score of 16, it showed agreeable sensitivity and specificity in detecting psychological distress [18]. Patients with K10 scores ≥16 were defined as having psychological distress. In this study, medication adherence was measured using the C-MMAS-8, with permission from the scale inventor Donald E. Morisky and licensure agreement for use. Informed consent was obtained from patients before each interview.

### 2.3. Psychometric Validation of the Chinese Version of the MMAS-8

The MMAS-8 is a general assessment and self-reported measure of medication-taking behavior, consisting of eight items measuring the failure of adherence when taking medication [13]. Item 1 asks “Do you sometimes forget to take your pills?” Item 2 asks “People sometimes miss taking their medications for reasons other than forgetting. Thinking over the past two weeks, were there any days when you did not take your medicine?” Item 3 asks “Have you ever cut back or stopped taking your medication without telling your doctor, because you felt worse when you took it?” Item 4 asks “When you travel or leave home, do you sometimes forget to bring along your medication?” Item 5 asks “Did you take your medicine yesterday?” Item 6 asks “When you feel like your illness is under control, do you sometimes stop taking your medicine?” Item 7 asks “Taking medication every day is a real inconvenience for some people. Do you ever feel hassled about sticking to your TB treatment plan?” Item 8 asks “How often do you have difficulty remembering to take all your medications?” Each item measures a specific medication-taking behavior. Item 5 is reverse worded. The responses for the items are yes/no except that the last item is on a five-point Likert scale. On the basis of the summated scores from the MMAS-8 ranging from 0 to 8, the scoring criteria can be obtained from the developer/owner of the scale and cut-points are predetermined by Morisky, adherence levels could be categorized as high (=8 points), medium (6 or 7 points) and low (<6 points) [8]. In this study, we focus on measuring level of low adherence; therefore, medication adherence was grouped as low-level and not low (medium/high) level.

The C-MMAS-8 psychometric properties were validated using reliability and validity. Reliability was measured in the form of the Cronbach’s alpha coefficients for both the corrected item-total correlation and if individual items were deleted. Factor analysis was used to measure the validity and the results were presented as the number of factors and the factor loadings between factor and each item. Eigenvalues >1 were used as a criterion to assess the number of factors, and items with factor loading >0.3 were considered significant.

### 2.4. Data Analysis

A database was set up using EpiData 3.1 (EpiData Association, Odense, Denmark) after data collection. First, psychometric validations in form of reliability and validity were determined for the C-MMAS-8. Second, adherence levels were described in relation to socio-demographic variables, clinical variables, and psychological status. At the same time, associations of the variables with medication adherence were tested using Pearson’s chi-square test. In order to keep as many variables as possible, variables with a *p*-value less than 0.2 were further analyzed in the next step of multivariate analysis. Third, multiple logistic regression was used to calculate the odds ratios (OR) and 95% confidence intervals (CI) of the variables related to adherence. Finally, the Receiver Operating Characteristic Curves (ROC) and the area under the ROC curve (AUC) were used to visualize and assess the prediction of the logistic regression model. The statistical significance was set at *p* < 0.05. Data were analyzed with the Statistical Analysis System (SAS 9.2 version) software (SAS Institute Inc., Cary, NC, USA).

## 3. Results

### 3.1. Sample Characteristics

There were 372 registered TB patients eligible in the sample frame being treated at home. Among these, 358 accessible TB patients participated in the survey, for a response rate of 96%. Of these, 70.9% of the surveyed patients were men, and 29.1% were women. The mean age was 54 years ranging from 15 to 96 years, and 42.1% of the patients were older than 60 years. As to the living situation, 21.8% of them were living without a partner or spouse. The patients had an average of six years of education. The mean length of treatment was nearly three months. Moreover, 68.7% of the patients ranked their household income below the local average.

### 3.2. Psychometric Properties of the C-MMAS-8

The mean score of the C-MMAS-8 in this study was 6.02 (Standard Deviation (SD) = 2.11) for the adherence scale. The total standardized Cronbach’s α as a measure of reliability was 0.80, which showed that the internal consistency of the scale was good. The corrected item-total correlations were >0.30 for most of the items composing the scale except for item 5, which was 0.21. When item 5 was deleted, the Cronbach’s α coefficient increased more than when other items were deleted (Table 1).

Further factor analysis indicated that the scale had two extracted factors with eigenvalues >1, and this was more obvious after varimax rotation. Factor 1 could explain 43.96% of the variance, and most items except item 5 had factor loadings >0.4. Factor 2 could explain 12.51% of the variance and the factor loadings of item 3, item 5, and item 6 were >0.3 (Table 2).

### 3.3. Level of Medication Adherence by Variables

According to the summated scores and referred cut-off point of the MMAS-8, 34.64% (124/358) of the TB patients were categorized as having low medication adherence, and 65.36% (234/358) as having medium/high adherence.

Although women, those of older age, those with low education, and those living without a partner had higher percentages of low medication adherence, none of these sociodemographic variables reached significance related to medication adherence in the chi-square test (*p* > 0.05) (Table 3). However, in Table 3 it can be seen that treatment length and psychological distress related significantly to adherence level, which may imply that longer treatment and having distress could lead to low adherence.

### 3.4. Factors Related to Low Adherence in the Logistic Regression Analysis

After testing for collinearity absence among the variables, all variables in chi-square tests with *p*-value < 0.2 were further analyzed in the multiple logistic regression. It was found that average age and above (OR = 2.28, 95% CI: 1.27–4.11) compared to below mean age, treatment reached or more than three months (OR = 2.75, 95% CI: 1.45–5.19) compared to that fewer than three months, and patients experiencing psychological distress (OR = 2.44, 95% CI: 1.46–4.07) compared to those with good mental status were significantly related to a low adherence level (Table 4). In Table 4, living without a partner (OR = 1.80, 95% CI: 1.00–3.25) compared to those living with a partner was weakly related to low adherence (*p* = 0.05). Other variables such as self-rated illness severity, self-reported limitations to daily life, treatment costs, and household income did not reach significance related to medication adherence in this study.

**Figure 1:**
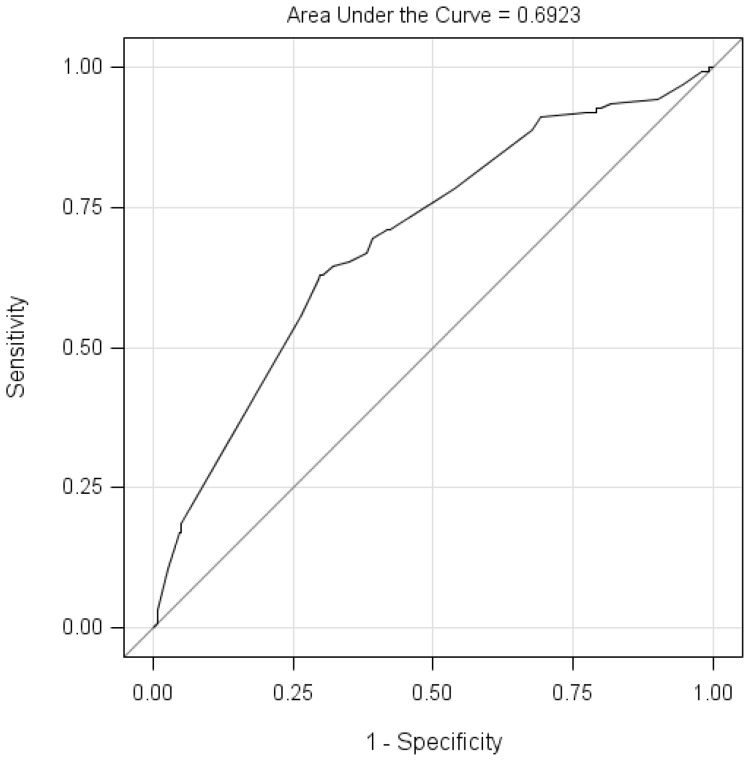
The Receiver Operating Characteristic Curve (ROC) for the logistic regression model. The ROC curve is a plot of sensitivity (events predicted to be events) versus 1-specificity (nonevents predicted to be events). The area under the ROC curve (AUC) could be used as a measure of goodness of fit.

The ROC and AUC were used to assess and visualize the predictive capability of the multiple logistic regression model. Figure 1 presented that the AUC was 0.6923 for the fitted model, and the sensitivity was 70.97% and the specificity was 58.12% when using the cutoff point (summated scores < 6) as the threshold for low adherence.

## 4. Discussion

Having a simple and inexpensive measurement instrument is of primary importance for many adherence studies. To our knowledge, this was the first time that the C-MMAS-8 was applied to an adherence study in rural TB patients in China. We adopted the language-validated version in order to keep the Chinese version consistent with its original English version, on the one hand, and to make the results comparable with other studies, on the other hand. In this study, the reliability measurements of the general Cronbach’s α coefficient and the coefficients of item-total correlations demonstrated high internal consistency. Item 5 “Did you take your medicine yesterday?” had weaker correlation with the total, which was consistent with some other studies [10,11,12]. As for the validity, the original and French MMAS-8 validation showed that the scale was uni-dimensional and had one single factor [8,12]. However, in this study two factors were extracted by factor analysis. Factor 1 had good loadings from almost all items, and was representative of general non-adherent behaviors. Factor 2 presented with good loadings from item 3, item 5, and item 6, which reflected intentional cutting back or stopping taking medicine when patients felt better due to quick relief of symptoms or felt worse because of side effects from the medication. This finding was consistent with an adherence study in type 2 diabetic patients in Thailand [11]. In all, and in spite of the different number of factors, the scale showed good reliability and validity in capturing the non-adherent behaviors of TB patients.

The scale showed that more than one-third (34.64%) of the TB patients had low medication adherence in rural areas, which is a situation that cannot be ignored when attempting to control TB. Further, in the univariate analysis using chi-square test, no significant association was found between socio-demographic variables and adherence level. However, in the multiple logistic regression, the variables of older age, treatment length, and depression were significantly related to low medication adherence. That older patients had lower medication adherence could be explained by their having poor memories and forgetting to take medication. However, one study of the French MMAS-8 and another study using C-MMAS-8 in Hong Kong reported lower adherence among younger adults with hypertension [12,19], while the original study of the MMAS-8 found age was not related to adherence level [8]. Therefore, more evidence for that association needs to be obtained in the future. Patients living without a partner had poorer medication adherence, which was confirmed by the results of some other studies [20,21]. Close family members, particular partners, play important roles in encouraging, supporting, and reminding patients to take their medication. However, this study only presented a weak association without statistical significance. In this study, patients with longer treatment duration (three months or more) had low medication adherence, and this suggests that adherence levels decrease over time, which has also been seen in some other studies [12,22,23]. However, it was found that hypertensive patients with shorter duration of treatment (five years or less) tended to have lower adherence than those with longer duration (10 years) in the Hong Kong study [19]. For TB patients, many patients feel better and tend to be less adherent to medication after the initial treatment stage, usually lasting three months. Depression exists as the comorbid condition in many patients with chronic diseases. We found that depression in TB patients was significantly related to a low level of medication adherence, and the fact that depression increased the risk of non-adherence to medication has been found in several other studies [24,25,26,27]. A meta-analysis showed that depressed patients had a three-fold higher risk of non-adherence than non-depressed patients [28]. This could suggest the importance of mental health promotion in medication adherence for chronic disease.

Although perceived illness severity could result in high adherence [29], self-rated illness severity and limitations on daily life were not related to adherence level in this study. The reason for this might be that these patients were being treated at home and might have had less severe symptoms than those being treated at a hospital. No significant relationship to adherence level was seen for household income level or treatment cost; this might be because the policy of providing free essential medications to TB patients could relieve the economic burden on patients. It appears that a lack of knowledge or health education in medication adherence instead of economic or financial obstacles is the main factor for low adherence in TB treatment in China [20,25].

The AUC was 0.69 when using the significant variables, which was acceptable for the multiple logistic regression model in predicting medication adherence. Moreover, with the cutoff of six points for low adherence, the model had a sensitivity of 70.97% in identifying low medication adherence and a specificity of 58.12% in detecting patients without low medication adherence. One adherence study using the Malaysian version of the MMAS-8 and laboratory test indices showed a sensitivity of 77.61% and specificity of 45.37% in diabetic patients [10]. In summary, the logistic regression model in this study has good predictive ability for medication adherence in TB patients.

The MMAS-8 has emphasized that self-reported non-adherence in medication is important and efficient when measuring medication-taking behaviors [15,30]. However, it would be more convincing if there were laboratory tests as comparative evidence, and every measurement method has its strengths and weakness. As for the low-adherence-related factors, associations from a cross-sectional study cannot be used to infer causality, thus the implications from this study need to be confirmed in a longitudinal study. Moreover, the level of TB knowledge among the patients could be an important variable for medication adherence, and the predictive model might be better if this had been taken into consideration; this is worth exploring in future studies.

## 5. Conclusions

This study showed that the C-MMAS-8 had good reliability and validity for measuring adherence levels in rural TB patients. The situation of low medication adherence was serious, and the factors associated with low adherence included older age, longer treatment (three months or more), and having depression. The predictive ability for low adherence was found to be acceptable by using the variables in a logistic model. Future interventions that target low adherence in rural TB patients could be modified to this particular population.

## Figures and Tables

**Table 1:** Reliability measurement of the C-MMAS-8.

Item	Corrected Item-Total Correlation Coefficient	Cronbach’s α If Item Was Deleted
Item 1	0.57	0.78
Item 2	0.61	0.77
Item 3	0.62	0.77
Item 4	0.55	0.78
Item 5	0.21	0.83
Item 6	0.61	0.77
Item 7	0.44	0.80
Item 8	0.57	0.78

Standardized Cronbach’s α = 0.80. C-MMAS-8: Chinese Version of the eight-item Morisky Medication Adherence Scale.

**Table 2:** Validity measurement of the C-MMAS-8.

Item	Factor 1 Loadings	Factor 2 Loadings
Item 1	0.78	−0.11
Item 2	0.74	0.11
Item 3	0.66	0.41
Item 4	0.72	−0.01
Item 5	0.03	0.92
Item 6	0.66	0.35
Item 7	0.54	0.17
Item 8	0.69	0.10
Eigenvalue	3.32	1.20
Proportion of variance explained	43.96%	12.51%

**Table 3:** Adherence level by sociodemographic variables, disease-related variables, and psychological status.

Variables	Total *N* = 358	Adherence Level		Chi-Square Test *p*-Value
	*n* (%)	Low *n* (%)	Medium/High *n* (%)	
Gender				0.22
Men	254 (70.95)	83 (32.68)	171 (67.32)	
Women	104 (29.05)	41 (39.42)	63 (60.58)	
Age				0.12
Average or above	208 (58.10)	79 (37.98)	129 (62.02)	
Below average	150 (41.90)	45 (30.00)	105 (70.00)	
School years				0.25
≥6	188 (52.51)	60 (31.91)	128 (68.09)	
<6	170 (47.49)	64 (37.65)	106 (62.35)	
Living status				0.06
With partner	280 (78.21)	90 (32.14)	190 (67.86)	
Without partner	78 (21.79)	34 (43.59)	44 (56.41)	
Household income				0.96
Average or above	112 (31.28)	39 (34.82)	73 (65.18)	
Below average	246 (68.72)	85 (34.55)	161 (65.45)	
Treatment length				0.005 ^*^
<3 months	76 (21.23)	16 (21.05)	60 (78.95)	
≥3 months	282(78.77)	108 (38.30)	174 (61.70)	
Cost				0.84
Below average	275 (76.82)	96 (34.91)	179 (65.09)	
Average or above	83 (23.18)	28 (33.73)	55 (66.27)	
Limitations to life				0.52
No/minor	278 (77.65)	92 (33.09)	186 (66.91)	
Moderate/severe	80 (22.35)	32 (40.00)	48 (60.00)	
Perceived severity				0.20
No/minor	91 (25.42)	29 (31.87)	62 (68.13)	
Moderate/severe	267 (74.58)	95 (35.58)	172 (64.42)	
Distress				0.002 *
No	122 (34.08)	29 (23.77)	93 (76.23)	
Yes	236 (65.92)	95 (40.25)	141 (59.75)	

* *p* < 0.05.

**Table 4:** Variables related to low medication adherence in multiple logistic regression analysis.

Variables	Low Adherence Level		*p*-Value
	OR	95% CI	
Age			0.0059
Below average	1	-	
Average or above	2.28 **	1.27–4.11	
Living status			0.0500
With partner	1	-	
Without partner	1.80	1.00–3.25	
Treatment length			0.0019
<3 months	1	-	
≥3 months	2.75 **	1.45–5.19	
Distress			0.0007
No	1	-	
Yes	2.44 ***	1.46–4.07	

* *p* < 0.05, ** *p* < 0.01, *** *p* < 0.001. OR: odds ratio; CI: confidence interval.
